# A Call for Research on the Prognostic Role of Follow-Up Histology in Celiac Disease: A Systematic Review

**DOI:** 10.3389/fphys.2019.01408

**Published:** 2019-11-19

**Authors:** Zsolt Szakács, Noémi Gede, Zoltán Gyöngyi, Margit Solymár, Dezső Csupor, Bálint Erőss, Áron Vincze, Alexandra Mikó, Andrea Vasas, László Szapáry, Dalma Dobszai, Viktória Balikó, Roland Hágendorn, Péter Hegyi, Judit Bajor

**Affiliations:** ^1^Institute for Translational Medicine, Medical School, University of Pécs, Pécs, Hungary; ^2^Szentágothai Research Centre, Medical School, University of Pécs, Pécs, Hungary; ^3^Department of Public Health Medicine, Medical School, University of Pécs, Pécs, Hungary; ^4^Department of Pharmacognosy, University of Szeged, Szeged, Hungary; ^5^Division of Gastroenterology, First Department of Medicine, Medical School, University of Pécs, Pécs, Hungary; ^6^Department of Interventional Cardiology, Heart Institute, University of Pécs, Pécs, Hungary

**Keywords:** celiac disease, mucosal recovery, persistent villous atrophy, follow-up biopsies, gluten-free diet

## Abstract

**Background:** Convincing evidence is lacking on the benefit of follow-up biopsy in celiac disease. Regardless, achieving mucosal recovery (MR) has remained a desirable goal of therapy. We aimed to conduct a systematic review to determine whether MR is a protective factor and persisting villous atrophy (PVA) has negative consequences on long-term outcomes of celiac disease.

**Methods:** Seven databases were searched for articles discussing celiac patients subjected to a gluten-free diet who had a follow-up biopsy, and clinical and laboratory characteristics were reported by follow-up histology (MR vs. PVA). Outcomes included clinical symptoms, mortality, malignant tumors, nutritional parameters, and metabolic bone disease. Comparative and descriptive studies were included. Since data proved to be ineligible for meta-analysis, the evidence was synthesized in a systematic review.

**Results:** Altogether, 31 studies were eligible for systematic review. Persisting symptoms were more frequently associated with PVA than with MR, although a lot of symptom-free patients had PVA and a lot of symptomatic patients achieved MR. PVA might be a risk factor of lymphomas, but mortality and the overall rate of malignant tumors seemed independent of follow-up histology. Patients with PVA tended to develop metabolic bone disease more often, although fracture risk remained similar in the groups except in hip fractures of which PVA was a risk factor. Reports on nutritional markers are only anecdotal.

**Conclusions:** The limited evidence calls for high-quality prospective cohort studies to be arranged to clarify the exact role of follow-up histology in celiac disease.

## Introduction

Celiac disease (CeD) is a systemic disorder with an increasing worldwide prevalence of ~1% (Di Sabatino and Corazza, 2009; Catassi et al., 2014). In CeD, the immune-mediated destruction of small intestinal villous architecture is triggered by dietary gluten (Reilly et al., 2017).

### Rationale of the Study

Although the value of intestinal biopsy at diagnosis is beyond dispute (Bai et al., 2013; Rubio-Tapia et al., 2013; Ludvigsson et al., 2014), the role of follow-up biopsy is a matter of controversy (Pekki et al., 2017). While the restitution of intestinal villi is expected on a strict gluten-free diet, the mucosa fails to restore entirely in a considerable fraction of patients (Szakacs et al., 2017). Recent guidelines recommend a follow-up biopsy if signs and symptoms persist or relapse despite strict adherence to a gluten-free diet (Bai et al., 2013; Rubio-Tapia et al., 2013; Ludvigsson et al., 2014). However, reports proposed that neither the resolution of symptoms (Bardella et al., 2007; Biagi et al., 2014; Fang et al., 2017; Mahadev et al., 2017) nor a strict dietary adherence (Szakacs et al., 2017) guarantees mucosal recovery (MR). One might expect that CeD patients with persistent villous atrophy (PVA) experience a less favorable disease course than those achieving MR (Haines et al., 2008), although convincing evidence is lacking. Yet, achieving MR has remained a desirable goal in CeD. The importance of the topic roots in the burden imposed by the endoscopic procedures and duodenal histological sampling as well as in the subsequent clinical decisions made upon follow-up histology.

### Objective of the Study

With this systematic review, we aimed to be the first who systematically collect all available evidence on the impact of follow-up histology (MR vs. PVA) on disease characteristics and clinical course of CeD.

### Research Question

Are CeD patients who have not achieved MR exposed to adverse clinical outcomes more frequently than those patients who have achieved MR?

## Methods

### Study Design

This systematic review was reported in accordance with the Preferred Reporting Items for Systematic reviews and Meta-Analyses 2009 (Moher et al., 2009).

### Participants and Exposure

Participants of this systematic review include CeD patients subjected to a gluten-free diet. The exposure of interest is the follow-up duodenal histology assessed as either MR or PVA.

### Systematic Review Protocol

The study protocol was registered *a priori* on PROSPERO under CRD42017069815.

### Search Strategy

We performed a systematic literature search in MEDLINE (via PubMed), Embase, Cochrane Controlled Register of Trials (CENTRAL), Web of Science, Scopus, WHO Global Index Medicus, and ClinicalTrials.gov from inception up to 14th September 2019 for relevant articles. Free-text terms and Medical Subject Headings were combined into a query, as follows: *celiac AND (“mucosal recovery” OR “mucosal healing” OR “mucosal atrophy” OR “intestinal atrophy” OR “duodenal atrophy” OR “villous atrophy” OR “persistent mucosal damage” OR “follow-up biopsy” OR “follow-up duodenal biopsy” OR “follow-up intestinal biopsy” OR “follow-up small intestinal biopsy” OR “follow-up histology” OR “repeated biopsy” OR “repeated histology” OR “control biopsy” OR “control histology”)*. No filters were imposed upon the search.

Relevant cited articles were explored by reviewing the reference lists of included papers. Citing articles were identified with Google Scholar.

### Data Sources, Study Selection, and Data Extraction

#### Eligibility Criteria

Eligible papers discussed CeD patients subjected to a gluten-free diet with an available record of duodenal follow-up histology, and reported on disease and patients characteristics by follow-up histology. Analytical and descriptive full-text articles or conference abstracts but not case studies were included without language restriction to reduce publication bias.

In our previous meta-analysis, we experienced a substantial variance in the definition of MR across studies (Szakacs et al., 2017). In this systematic review, we defined MR as the resolution of villous atrophy at follow-up biopsy assessed as “non-atrophic” histology based on crypt height:villous depth ratio, Marsh grades 0-2, Marsh-Oberhuber grades 0-2, or Corazza-Villanacci grade A; while PVA was defined as “atrophic” histology, Marsh grade 3, Marsh-Oberhuber grade 3, or Corazza-Villanacci grade B1-B2 (Marsh, 1992; Oberhuber et al., 1999; Corazza and Villanacci, 2005).

Disease and patients characteristics included signs and symptoms, vitamin and mineral levels, anemia, body mass index, metabolic bone disease, malignant tumors and other co-morbid conditions, and long-term mortality.

#### Selection and Data Collection

Records were combined in a reference manager software (EndNote X7.4, Clarivate Analytics, Philadelphia, PA, the USA) to remove duplicates and overlapping database content. Then, the standard three-step selection was performed by title, abstract and full-texts. Each step was carried out by two investigators in duplicate. Discrepancies were resolved by third-party arbitration. K-statistics was used to measure the agreement between the investigators after each step.

Numeric and text data were extracted by two investigators onto a pre-defined Excel sheet, discrepancies were resolved by consensus. Although we contacted the authors of original studies for further raw data via email, we discarded these data from the systematic review when we realized that the material is ineligible for meta-analysis due to several reasons (as detailed later).

#### Design of the Studies Included and Quality Assessment

First, the design of the included papers was identified. Then, quality indicators were chosen based on the Quality in Prognostic Studies tool (Hayden et al., 2006), as follows:

- way of recruitment,
- diagnosis of CeD (only biopsy-verified cases or not),
- the recency of diagnosis (newly diagnosed patients or treated patients were included),
- representativeness of study population to the general CeD population (based on the inclusion and exclusion criteria of the individual studies),
- timing of follow-up biopsy (taken prospectively after enrolment or earlier),
- time elapsed between the diagnosis of CeD and the follow-up biopsy, and that between the follow-up biopsy and the measurement of outcomes,
- definition of PVA (histological classification),
- biopsy sampling site,
- timing of outcome assessment (prospectively after enrolment or earlier),
- definitions of outcomes (with cut-off values if applicable),
- blinding,
- adherence to a gluten-free diet (strict or not), and
- statistical considerations (the analysis directly compared the clinical characteristics by MR and PVA or not and the analysis was adjusted for reasonable confounding factors or not).

## Results

### Study Selection and Characteristics

**Figure 1:**
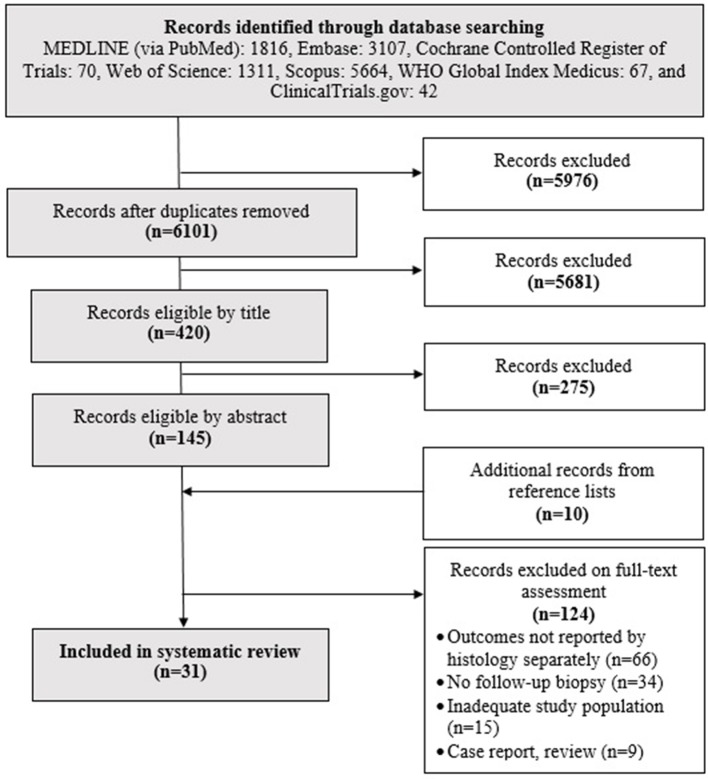
Flowchart of the search and selection process.

After careful search and selection (Figure 1), 31 papers were included in the systematic review (Souroujon et al., 1982; Thornquist et al., 1993; Valdimarsson et al., 1994; Walters et al., 1995; Kemppainen et al., 1998; Selby et al., 1999; Ciacci et al., 2002; Bardella et al., 2007; Cammarota et al., 2007; Kaukinen et al., 2007; Carroccio et al., 2008; Dickey et al., 2008; Koskinen et al., 2010; Rubio-Tapia et al., 2010; Tuire et al., 2012; Lebwohl et al., 2013a,b, 2014, 2015a,b; Ghazzawi et al., 2014; Pekki et al., 2015, 2017; Cornell et al., 2016; Haere et al., 2016; Fang et al., 2017; Leonard et al., 2017; Mahadev et al., 2017; Emilsson et al., 2018; Kurien et al., 2018; Ludvigsson et al., 2018) (Table 1). Cohen's κ representing inter-reviewer agreement was 0.72 (substantial), 0.79 (substantial), and 0.92 (almost perfect) for selection by title, abstract, and full-text; respectively.

**Table 1:** Characteristics of the studies included.

**Study**	**Setting**	**Study population and design**						**Definition of villous atrophy and sampling site**
		**Recruitment**	**Age**	**Biopsy-proven CeD?**	**Representative population?^**a**^**	**Newly diagnosed CeD?**	**Prospectively taken follow-up biopsy?**	
Bardella et al. (2007)	Italy (single-center)	Consecutive pts	Adults and children	Yes	No	No	No	Marsh-Oberhuber 3_a−c_, duodenum
Cammarota et al. (2007)	Italy (2-center)	Consecutive pts	Adults	Yes	Yes	No	Yes	Marsh-Oberhuber 3_a−c_, duodenum
Carroccio et al. (2008)	Italy (single-center)	Consecutive symptomatic pts and randomly selected matched asymptomatic pts	Adults	Yes	Yes	No	Yes	Marsh-Oberhuber 3_a−c_, duodenum
Ciacci et al. (2002)	Italy (single-center)	Consecutive pts	Adults	Yes	Yes	No	No	Marsh 3, small intestine
Cornell et al. (2016)	Poland (single-center)	Consecutive volunteer	Adults	Yes	Yes	No	Yes	Marsh 3, duodenum
Dickey et al. (2008)	Northern Ireland (single-center)	Consecutive pts	Adults	Yes	Yes	No	Yes	Marsh 3, duodenum
Fang et al. (2017)	The USA (Single-center)	Consecutive pts	Adults	(?)	No	No	No	Corazza-Villanacci B_1−2_, duodenum
Ghazzawi et al. (2014)	The USA (single-center)	Consecutive pts	Children	Yes	Yes	No	No	Marsh 3, small intestine
Haere et al. (2016)	Norway (single-center)	Consecutive pts by invitation	Adults	Yes	Yes	No	Yes	Marsh-Oberhuber 3_a−c_, duodenum
Kaukinen et al. (2007)	Finland (single-center)	Consecutive pts with persistent villous atrophy and randomly selected pts with mucosal recovery	Adults	(?)	Yes	No	No	Vh/Cr<2, duodenum, jejunum
Kemppainen et al. (1998)	Finland (single-center)	*Post-hoc* analysis of a randomized trial	Adults	Yes	Yes	No	No	Not reported (“atrophic”), duodenum
Koskinen et al. (2010)	Finland (single-center)	Consecutive pts	Adults and children	Yes	No	Yes/No^b^	Yes	Vh/Cr<2, jejunum
Lebwohl et al. (2013a,b); Lebwohl et al. (2014, 2015a,b), Emilsson et al. (2018), Ludvigsson et al. (2018), Kurien et al. (2018)	Sweden (28-center)	Consecutive pts (nationwide sample)	Adults and children	Yes	Yes	No	No	Marsh 3, small intestine
Leonard et al. (2017)	Italy (2-center)	Consecutive pts	Children	Yes	Yes	No	No	Marsh-Oberhuber 3_a−c_, duodenum
Mahadev et al. (2017)	North-America, Ireland, UK, Norway, and Finland (multicenter)	*Post-hoc* analysis of a randomized trial	Adults	(?)	No	No	No	Vh/Cr≤2, duodenum
Pekki et al. (2015)	Finland (single-center)	Consecutive pts	Adults	Yes	Yes	(?)	Yes	Vh/Cr<2, duodenum
Pekki et al. (2017)	Finland (single-center)	Consecutive volunteers	Adults	Yes	Yes	No	No	Vh/Cr (“atrophic”), small intestine
Rubio-Tapia et al. (2010)	The USA (single-center)	Consecutive pts	Adults	Yes	Yes	No	No	Vh/Cr<3, duodenum
Selby et al. (1999)	Australia (single-center)	Symptomatic and asymptomatic pts from a previous food-intolerance study	Adults	Yes	Yes	No	No	Vh/Cr<2, duodenum
Souroujon et al. (1982)	Israel (single-center)	Consecutive pts	Adults and Children	Yes	Yes	Yes	Yes	Not reported (“atrophic”), small intestine
Thornquist et al. (1993)	Norway (single-center)	Consecutive pts by invitation	Adults	Yes	Yes	No	Yes/No^c^	Alexander III-IV, jejunum
Tuire et al. (2012)	Finland (single-center)	Consecutive volunteers	Adults	Yes	Yes	No	Yes	Marsh 3, duodenum
Valdimarsson et al. (1994)	Sweden (single-center)	Consecutive pts with persistent villous atrophy and matched pts with mucosal recovery	Adults	(?)	Yes	No	No	Alexander III-IV, small intestine
Walters et al. (1995)	UK (single center)	Consecutive pts by invitation	Adults	Yes	No	No	Yes/No^c^	Not reported (“atrophic”), small intestine

a Study population is considered representative to the average CeD population if the study avoided inappropriate exclusions.

b The study observed a group of newly diagnosed CeD patients for the short-term and a group of followed-up patients for the long-term.

c Not all patients were newly diagnosed.

*(?) indicates uncertainty. CeD, celiac disease; pts, patients; Vh/Cr, villous height/crypt depth ratio*.

Out of the 31 papers, eight retrieved data on different outcomes from the Swedish CeD registry; therefore, study populations partially overlap (Lebwohl et al., 2013a,b, 2014, 2015a,b; Emilsson et al., 2018; Kurien et al., 2018; Ludvigsson et al., 2018).

### Highlights of the Last Five Years

In this subchapter, we summarize the findings of the studies which investigated the prognostic role of follow-up histology and were published after January 2015 (Lebwohl et al., 2013a, 2015a; Pekki et al., 2015, 2017; Haere et al., 2016; Fang et al., 2017; Mahadev et al., 2017; Emilsson et al., 2018; Kurien et al., 2018; Ludvigsson et al., 2018). Eight studies were conducted in Scandinavia, one in the USA, and there was another multicenter study from Europe and North-America. Evidence from univariate analysis suggested a borderline association of PVA with persisting symptoms (OR = 1.656 with CI: 0.949–2.889; *p* = 0.076 favoring mucosal recovery) (Fang et al., 2017), which was not confirmed by co-variate-adjusted analysis of the baseline cohort of patients of a multicenter randomized trial on symptomatic CeD patients (Mahadev et al., 2017). Conclusions from three Scandinavian studies on symptom and well-being scores corroborated these findings (Pekki et al., 2015, 2017; Haere et al., 2016). PVA might be associated with lower lumbar T-score measured at 1 year after diagnosis, although neither the risk of osteoporosis at 5 years after diagnosis nor that of fractures at 2 years after diagnosis was higher in this group (Pekki et al., 2015, 2017). Decreased vitamin D and calcium levels might contribute to the impaired bone mineral density (Fang et al., 2017). Regarding other nutrients and minerals, zinc deficiency should be highlighted (39 vs. 14% in patients with PVA and MR, respectively; *p* = 0.0005). The effect of PVA did not manifest itself regarding erythropoiesis: three studies reported similar hemoglobin levels and rate of anemia in patients with PVA and MR (Pekki et al., 2015; Fang et al., 2017; Mahadev et al., 2017). Rate of malignant tumors and that of lymphomas were not significantly different in histology groups in two studies 5 and 8 years after diagnosis (Pekki et al., 2015, 2017). Similar neutral associations were found on the rate of cardiovascular diseases (Lebwohl et al., 2015a; Mahadev et al., 2017), serious infections including sepsis, streptococcal, pneumococcal, influenza, herpes zoster, and *Clostridium difficile* infections (Emilsson et al., 2018), and adverse pregnancy outcomes (Lebwohl et al., 2015b). MR seemed to be a protective factor against respiratory and dermatological diseases (Pekki et al., 2017). In contrast, anxiety and depression co-occurred more frequently with MR (Ludvigsson et al., 2018) as did epilepsy (Kurien et al., 2018). With respect to overall mortality, a higher rate was reported with PVA on 8-years follow-up (14 vs. 9%) but the difference was not statistically significant (*p* = 0.259).

### Narrative Review

In this subchapter, we summarize all available evidence published on the effect of MR and PVA on the outcomes of CeD.

#### Symptoms, Symptom Scores and Quality of Life

In asymptomatic patients, PVA rates ranged from 2 to 62% (Walters et al., 1995; Bardella et al., 2007; Koskinen et al., 2010; Cornell et al., 2016). In symptomatic patients, PVA rate was 38% (Mahadev et al., 2017) (Table 2). Six studies reported symptoms as dichotomies (symptomatic vs. asymptomatic) (Thornquist et al., 1993; Selby et al., 1999; Kaukinen et al., 2007; Carroccio et al., 2008; Fang et al., 2017; Leonard et al., 2017). In univariate analysis, the percentage points of symptomatic patients with PVA and MR were 55 vs. 70% (*p* = NS) (Leonard et al., 2017), 69 vs. 57% (*p* = 0.076) (Fang et al., 2017), and 80 vs. 25% (*p* < 0.0001) (Carroccio et al., 2008), respectively (Table 3). Only one study adjusted for reasonable co-variates where the adjusted OR was 3.2, 95% CI: 1.6–6.4 (*p* = 0.001), indicating that patients with PVA tended to be symptomatic more often (Carroccio et al., 2008). Regarding the individual symptoms, only one study adjusted for co-variates where none of the symptoms in focus, that is, diarrhea, bloating, abdominal pain, nausea, tiredness, constipation, heartburn and headache, was significantly associated with PVA (Mahadev et al., 2017) (Table 3).

**Table 2:** Persistent villous atrophy in studies including only symptomatic or asymptomatic celiac patients.

**Study**	**Symptoms assessed prospectively?**	**Symptoms assessed at the time of follow-up biopsy?**	**Only strict GFD?**	**Symptoms**	**N^**0**^ of pts with persistent villous atrophy/total** **(% of total)**	**Timing of follow-up biopsy** **(from CeD diagnosis)**
**ASYMPTOMATIC POPULATION**						
Bardella et al. (2007)	No	Yes	Yes	Not specified	71 cases/114 pts (62)	2 years (median) with 1–23 years (range)
Walters et al. (1995)	(?)	(?)	Yes	Not specified	9 cases/17 pts (53)	2 years (min)
Koskinen et al. (2010)	Yes	Yes	(?)	Specified^a^	21 cases/71 pts (30)	1 years
	Yes	Yes	(?)		2 cases/105 pts (2)	8 years (median) with 2–41 years (range)
Cornell et al. (2016)	Yes	Yes	(?)	Not specified	9 cases/20 pts (45)	4 m-36 years (range)
**SYMPTOMATIC POPULATION**						
Mahadev et al. (2017)	No	Yes	Yes	Specified^b^	511 cases/1,345 pts (38)	4 years (median) with 1 years (min)

a Symptoms included abdominal complaints, malabsorption, and extraintestinal symptoms.

b *Symptoms include bloating, tiredness, abdominal pain, diarrhea, nausea, constipation, heartburn, and headache. (?) indicates uncertainty. CeD, celiac disease; GFD, gluten-free diet; m, month; min, minimum; pts, patients*.

**Table 3:** Presence of symptoms in celiac patients with persistent villous atrophy and mucosal recovery.

**Study**	**Symptoms assessed prospectively?**	**Symptoms assessed at the time of follow-up biopsy?**	**Only strict GFD?**	**Symptoms**	**N^**0**^ of symptomatic pts/total with persistent villous** **atrophy (% of total)**	**N^**0**^ of symptomatic pts/total with mucosal recovery** **(% of total)**	**Statistics** **(atrophy vs. recovery)**	**Timing of follow-up biopsy** **(from CeD diagnosis)**
**SYMPTOMATIC vs. ASYMPTOMATIC PTS**								
Carroccio et al. (2008)	Yes	Yes	Yes	Specified^a^	36 cases/45 pts (80)	6 cases/24 pts (25)	OR = 12 (CI: 3.7–38.9) (*p* < 0.0001, favors recovery); **adjusted OR** **=** **3.2** **(CI: 1.6–6.4)** **(*p*** **=** **0.001, favors recovery)**^***b***^	6.5 y and 6.1 y (means for groups) with 3.6 y and 4.6 y (SDs for groups)
Fang et al. (2017)	No	Yes	Yes	Specified^c^	53 cases/77 pts (69)	116 cases/203 pts (57)	OR = 1.656 (CI: 0.949–2.889) (*p* = 0.076, favors recovery)	2 y (min)
Kaukinen et al. (2007)	Yes	No^d^	Yes	Not specified	4 cases/13 pts (31)	Not reported	Not reported	8 y and 10 y (medians for groups) with 3–30 y (range)
Leonard et al. (2017)	No	Yes	No	Not specified	11 cases/20 pts (55)	58 cases/83 pts (70)	*p* = NS	2.4 y (median) with 1–12 y (range) and 1.4–4 y (Q_1_-Q_3_)
Selby et al. (1999)	(?)	No^e^	Yes	Not specified	5 cases/26 pts (19)	3 cases/24 pts (13)	Not reported	Not reported
Thornquist et al. (1993)	Yes	No^e^	Yes	Specified^f^	1 case/4 pts (25)	7 cases/9 pts (78)	Not reported	17 y (mean) with 15–18 y (range?)
**SPECIFIC SYMPTOMs (PRESENT vs. ABSENT)**								
Cammarota et al. (2007)	Yes	Yes	No	Diarrhea	10 cases/49 pts (20)	2 cases/16 pts (13)	Not reported	6–14 m (range)
				Bloating	9 cases/49 pts (18)	3 cases/16 pts (19)		
				Abdominal pain	5 cases/49 pts (10)	7 cases/16 pts (44)		
Carroccio et al. (2008)	Yes	Yes	Yes	Typical symptoms	6 cases/45 pts (13)	0 cases/24 pts (0)	*p* = 0.005 (favors recovery)	6.5 y and 6.1 y (means for groups) with 3.6 y and 4.6 y (SDs for groups)
				GERD-like symptoms	9 cases/45 pts (20)	3 cases/24 (13)	*p* = 0.01 (favors recovery)	
				lower abdominal symptoms	21 cases/45 pts (47)	3 cases/21 pts (14)	*p* = 0.001 (favors recovery)	
Ghazzawi et al. (2014)	No	Yes	Yes	Abdominal pain	2 cases/5 pts (40)	17 cases/34 pts (50)	Not reported	2 y (mean) with 22 m (SD) and 4–120 m (range)
				Diarrhea	2 cases/5 pts (40)	5 cases/34 pts (15)		
				Constipation	0 cases/5 pts (0)	5 cases/34 pts (15)		
Mahadev et al. (2017)	No	Yes	Yes	Bloating	431 cases/511 pts (84)	736 cases/834 pts (88)	*p* = 0.04 (favors atrophy), *p* = 0.001 (favors atrophy), *p* = 0.0009 (favors atrophy), and *p* = 0.04 (favors recovery) for bloating, abdominal pain, nausea, and heartburn, respectively; *p* = NS for the other comparisons; **adjusted** ***p*** **=** **NS for all comparisons**^***g***^	4 y (median) with 1 y (min)
				Abdominal pain	410 cases/511 pts (80)	724 cases/834 pts (87)		
				Tiredness	421 cases/511 pts (82)	708 cases/834 (85)		
				Diarrhea	375 cases/511 pts (73)	643 cases/834pts (77)		
				Nausea	239 cases/511 pts (47)	451 cases/834 pts (54)		
				Constipation	263 cases/511 pts (51)	421 cases/834 pts (50)		
				Heartburn	140 cases/511 pts (27)	186 cases/834 pts (22)		
				Headache	112 cases/511 pts (22)	208 cases/834 pts (25)		

Multivariate analyses are highlighted with bold.

a Symptoms included typical (chronic diarrhea, weight loss, anemia), GERD-like, and lower abdominal symptoms (abdominal pain, constipation).

b Analysis was adjusted for age, gender, duration of GFD, diagnostic histological severity.

c Symptoms included abdominal pain, bloating, nausea, fatigue, diarrhea, bloody stool, steatorrhea, and weight loss.

d Symptoms were assessed 4–5 years after the follow-up biopsy.

e Symptoms were evaluated after follow-up biopsy (interval undetermined).

f Symptoms included gastrointestinal and malabsorptive symptoms, such as diarrhea, borborygmi, abdominal pain, fatty stool, and anemia.

g *The analysis was adjusted for age, gender, body mass index, duration of GFD, medications, and laboratory tests. (?) indicates uncertainty. CeD, celiac disease; CI, confidence interval; GERD, gastroesophageal reflux disease; GFD, gluten-free diet; m, month; min, minimum; OR, odds ratio; pts, patients; NS, non-significant; SD, standard deviations; Q_1_-Q_3_, 25 and 75% quartiles; y, year*.

Five studies (Kaukinen et al., 2007; Tuire et al., 2012; Pekki et al., 2015, 2017; Haere et al., 2016) reported gastrointestinal symptom scores measured with the Gastrointestinal Symptom Rating Scale (GSRS) or its irritable bowel syndrome-adapted version (GSRS-IBS). Four of which performed statistical analysis and reported no association between histology and the scores consistently (Kaukinen et al., 2007; Pekki et al., 2015, 2017; Haere et al., 2016) (Table 4). Results were similar in quality of life measured with the Psychological General Well-Being Index (PGWB) and/or the 36-Item Short Form Health Survey (SF-36) (Kaukinen et al., 2007; Tuire et al., 2012; Pekki et al., 2015, 2017) (Table 4).

**Table 4:** Symptom scores and quality of life indices in celiac patients with persistent villous atrophy and mucosal recovery.

**Study**	**Symptoms assessed prospectively?**	**Symptoms assessed at the time of follow-up biopsy?**	**Only strict GFD?**	**Tool**	**Persistent villous atrophy**		**Mucosal recovery**		**Statistics (atrophy vs. recovery)**	**Timing of follow-up biopsy (from CeD diagnosis)**
					**N^**0**^ of pts**	**Score**	**N^**0**^ of pts**	**Score**		
**GASTROINTESTINAL SYMPTOM SCORES**										
Pekki et al. (2017)	Yes	No^a^	No	GSRS	200	Not reported	276	Not reported	*p* = NS	1 y
Kaukinen et al. (2007)	Yes	Yes	Yes	GSRS	13	mean: 2.0 (CI: 1.4–2.6)	18	mean: 2.0 (CI: 1.7–2.3)	*p* = NS	8 y and 10 y (medians for groups) with 3–30 y (range)
Pekki et al. (2015)	Yes	Yes	No	GSRS	85	Median: 1.5 (Q_1_-Q_3_: 1.3–1.8)	178	Median: 1.5 (Q_1_-Q_3_: 1.3-2.0)	*p* = 0.321	1 y
		No^a^	No	GSRS	∑44^b^	Median: 1.6 (Q_1_-Q_3_: 1.0–4.0)	∑44^b^	Median: 1.9 (Q_1_-Q_3_: 1.0–3.0)	*p* = 0.132	5 y (median)
Tuire et al. (2012)	Yes	Yes	Yes	GSRS^c^	7	Mean: 1.73 (CI: 1.39-2.07)	170	Not reported	Not reported	2–41 y (range)
Haere et al. (2016)	Yes	Yes	No	GSRS-IBS^d^	7	Mean: 25.1 (*SD* 9.9)	116	Mean: 26.4 (*SD*: 9.9)	*p* = NS	9.3 y (mean) with 5 y (*SD*) and 2 y (min)
**QUALITY OF LIFE SCORES**										
Pekki et al. (2017)	Yes	No^a^	No	PGWB	200	Not reported	276	Not reported	*p* = NS	1 y
				SF-36					*p* = NS	
Kaukinen et al. (2007)	Yes	Yes	Yes	PGWB	13	Mean: 101 (CI: 94-108)	18	Mean: 101 (CI: 92-110)	*p* = NS	8 y and 10 y (medians for groups) with 3–30 y (range)
Pekki et al. (2015)	Yes	Yes	No	PGWB	85	Median: 112 (Q_1_-Q_3_: 102–118)	178	Median: 111 (Q_1_-Q_3_: 100–117)	*p* = 0.699	1 y
		No^a^	No	PGWB	∑44^b^	median: 109 (Q_1_-Q_3_: 52–124)	∑44^b^	Median: 108 (Q_1_-Q_3_: 62–121)	*p* = 0.416	5 y (median)
Tuire et al. (2012)	Yes	Yes	Yes	PGWB^b^	7	Mean: 112.7 (CI: 110.6–114.8)	170	Not reported	Not reported	2–41 y (range)

a Scores were assessed years after the follow-up biopsy.

b The number of patients were not reported for atrophic and recovery groups separately.

c Subdimensions of GSRS were reported within the original article in detail.

d *A 3-day modified version of the original tool was used. CeD, celiac disease; CI, confidence interval; GFD, gluten-free diet; GSRS, Gastrointestinal Symptom Rating Scale; GSRS-IBS: Gastrointestinal Symptom Rating Scale for Irritable Bowel Syndrome; min, minimum; NS, non-significant; PGWB, Psychological General Well-Being; pts, patients; SF-36, Short Form Health Survey-36; Q_1_-Q_3_, 25% and 75% quartiles; SD: standard deviation*.

#### Metabolic Bone Disease and Bone Fractures

Four studies assessed bone mineral density in univariate analysis (Valdimarsson et al., 1994; Walters et al., 1995; Kaukinen et al., 2007; Pekki et al., 2015) (Table 5). Patients with PVA tended to have lower forearm, femoral and trochanter Z-scores (Valdimarsson et al., 1994); and lower femoral T-score with similar femoral Z- and lumbar T- and Z-scores than those with MR (Pekki et al., 2015). One study showed a reduced bone mineral density with PVA (OR = 24.5, *p* < 0.0275 favoring MR) (Walters et al., 1995). Two studies investigated the risk of osteoporosis in the long-term and reported conflicting results: a case-control study reported an increased frequency of osteoporosis with PVA (Kaukinen et al., 2007) but a cohort study failed to confirm (Pekki et al., 2015).

**Table 5:** Bone mineral density in celiac patients with persistent villous atrophy and mucosal recovery.

**Study**	**Parameter of interest assessed prospectively?**	**Parameter of interest assessed at the time of follow-up biopsy?**	**Only strict GFD?**	**Bone mineral density**	**Persistent villous atrophy**		**Mucosal recovery**		**Statistics (atrophy vs. recovery)**	**Timing of follow-up biopsy** **(from CeD diagnosis)**
					**Scores**	**N^**0**^ of pts**	**Scores**	**N^**0**^ of pts**		
**T- AND Z-SCORES**										
Pekki et al. (2015)	Yes	Yes	No	Lumbar (T-score)	Median: −1.1 (Q_1_-Q_3_: −2.2 to−0.2)	∑152^a^	Median: −0.7 (Q_1_-Q_3_: −1.7 to 0.4)	∑152^a^	*p* = 0.117	1 y
				Femoral neck (T-score)	Median: −1.1 (Q_1_-Q_3_: −1.9 to−0.5)	85 pts	Median: −0.6 (Q_1_-Q_3_: −1.5 to 0.0)	178 pts	*p* = 0.024 (favors recovery)	
				Lumbar (Z-score)	Median: −0.5 (Q_1_-Q_3_: −1.3 to −0.1)	∑159^a^	Median: 0.1 (Q_1_-Q_3_: −1.1 to 0.9)	∑159^a^	*p* = 0.376	
				Femoral neck (Z-score)	Median: −0.5 (Q_1_-Q_3_: −0.7 to −0.1)	∑140^a^	Median: −0.3 (Q_1_-Q_3_: −0.6 to 0.3)	∑140^a^	*p* = 0.323	
Valdimarsson et al. (1994)	Yes	(?)	No	Forearm (Z-score)	Not reported	13 pts	Not reported	17 pts	*P* < 0.01 (favors recovery)	8 y and 9 y (medians for groups) with 4–14 y (range)
				Femoral neck (Z-score)					*p* < 0.01 (favors recovery)	
				Femoral trochanter (Z-score)					*p* < 0.05 (favors recovery)	
					**Affected pts/total with persistent villous atrophy (% of total)**		**Affected pts/total with mucosal recovery (% of total)**			
**OSTEOPOROSIS AND OSTEOPENIA**										
Kaukinen et al. (2007)	Yes	Yes	Yes	Osteoporosis (T-score ≤−2.5 *SD*)	7 cases/12 pts (58)		4 cases/18 pts (22)		*p* = 0.04	8 y and 10 y (medians for groups) with 3–30 y (range)
				Osteopenia (T-score between −1.0 and −2.4 *SD*)	4 cases/12 pts (33)		8 cases/18 pts (44)		*p* = NS	
Pekki et al. (2015)	Yes	No^b^	No	Osteoporosis (T- and Z-scores)	8 cases/71 pts (14)		16 cases/134 pts (13)		*p* = 0.850	5 y (median)
Walters et al. (1995)	Yes	No^b^	Yes	Osteopenia (Z-score)	7 cases/9 pts (78)		1 case/8 pts (13)		*p* < 0.0275 (favors recovery)	2 y (min)

All measurements were performed with dual-energy X-ray absorptiometry except in forearm bone mineral density in the study of Valdimarsson et al. (1994) which used single-photon absorptiometry.

a The number of patients were not reported for atrophic and recovery groups separately.

b *Parameter was assessed years after follow-up biopsy. (?) indicates uncertainty. GFD, gluten-free diet; m, month; min, minimum; NS, non-significant; pts, patients Q_1_-Q_3_, 25% and 75% quartiles; SD, standard deviation; y, year*.

Three studies (Valdimarsson et al., 1994; Lebwohl et al., 2014; Pekki et al., 2017) reported no association between fractures and follow-up histology, except in the frequency of hip fractures being seemingly increased after a 5-years follow-up with PVA (adjusted HR = 2.18, CI: 1.17–4.05) (Lebwohl et al., 2014) (Table 6).

**Table 6:** Fractures in celiac patients with persistent villous atrophy and mucosal recovery.

**Study**	**Events assessed prospectively?**	**Only strict GFD?**	**Type of fracture**	**Persistent villous atrophy**	**Mucosal recovery**	**Statistics (atrophy vs. recovery)**	**Timing of follow-up biopsy** **(from CeD diagnosis)**	**Follow-up period**
Valdimarsson et al. (1994)	Yes	No	Any fracture	9 fractures in 6 cases/13 pts	5 fractures in 5 cases/17 pts	*p* = NS for the number of patients with fractures	8 y and 9 y (medians for groups) with 4–14 y (range)	Not reported
Pekki et al. (2017)	Yes	No	Any fracture	53 cases/200 pts	80 cases/276 pts	*p* = 0.859	1 y	Years (?)
Lebwohl et al. (2014)	No	(?)	Any fracture^a^	492 cases/34409 PYO	483 cases/36418 PYO	**HR** **=** **0.93 (CI: 0.82–1.06)**^**b**^	6 m−5 y (range)	8.6 y (median) with 6.9–15.7 y (Q_1_-Q_3_) from follow-up biopsy
			Likely osteoporotic fracture^a^	124 cases/20617 PYO	96 cases/11916 PYO	**HR** **=** **1.11 (CI: 0.84-1.46)**^**b**^		
			Hip fracture^a^	26 cases/37925 PYO	63 cases/39851 PYO	**HR** **=** **1.67 (CI: 1.05–2.66)**^**b**^ **(favors recovery)**		

Multivariate analyses are highlighted with bold.

a Detailed data are available in the original article.

b *The analysis was adjusted for age, gender, duration of celiac disease, calendar period, and educational attainment. (?) indicates uncertainty. CI, confidence interval; GFD, gluten-free diet; HR, hazard ratio (reference group: mucosal recovery); NS, non-significant; PYO, person-years observation; Q_1_-Q_3_: 25 and 75% quartiles*.

#### Micro- and Macronutrients

Table 7 summarizes the laboratory findings.

**Table 7:** Vitamins, minerals, and homocysteine in celiac patients with persistent villous atrophy and mucosal recovery.

**Study**	**Parameter of interest assessed prospectively?**	**Parameter of interest assessed at the time of follow-up biopsy?**	**Only strict GFD?**	**Laboratory study**	**Persistent villous atrophy**			**Mucosal recovery**			**Statistics (atrophy vs. recovery)**	**Timing of follow-up biopsy (from CeD diagnosis)**
					**Total N^**0**^ of pts**	**N^**0**^ of pts with deficiency (% of total)**	**Level**	**Total N^**0**^ of pts**	**N^**0**^ of pts with deficiency (% of total)**	**Level**		
**VITAMIN B**_**2**_ **(RIBOFLAVIN)**												
Dickey et al. (2008)	Yes	Yes	Yes	EGRAC	24		Mean: 1.38 (*SD*: 0.19)	41		Mean: 1.28 (SD: 0.13)	Not reported	1 y (min)
**VITAMIN B**_**6**_ **(PYRIDOXINE)**												
Dickey et al. (2008)	Yes	Yes	Yes	Plasma pyridoxal phosphate (nmol/l, vitamin)	24		Mean: 69.9 (*SD*: 24.0)	41		Mean: 91.0 (*SD*: 43.3)	Not reported	1 y (min)
**VITAMIN B**_**9**_ **(FOLIC ACID)**												
Dickey et al. (2008)	Yes	Yes	Yes	Erythrocyte folic acid (nmol/l)	24		Mean: 900 (*SD*: 574)	41		Mean: 1048 (*SD*: 791)	Not reported	1 y (min)
Dickey et al. (2008)	Yes	Yes	Yes	Serum folic acid (nmol/l)	24		Mean: 23.5 (SD: 22.8)	41		Mean: 25.7 (SD: 31.5)	Not reported	1 y (min)
Kemppainen et al. (1998)	No	Yes	(?)	Erythrocyte folic acid *(cut-off: not reported)*	37	3 (8)		3	0 (0)		Not reported	1 y
Pekki et al. (2015)	Yes	Yes	No	Erythrocyte folic acid (nmol/l)	∑142^a^		Median: 548 (Q_1_-Q_3_: 424–676)	∑142^a^		Median: 508 (Q_1_-Q_3_: 383–650)	*p* = 0.547	1 y
Thornquist et al. (1993)	Yes	No^b^	No	Serum folic acid	4		Not reported^d^	9		Not reported^d^	Not reported	17 y (mean) with 15–18 y (range ?)
Tuire et al. (2012)	Yes	Yes	Yes	Erythrocyte folic acid (nmol/l)	7		Median: 486 (range: 243–1,849)	170		Not reported	Not reported	2–41 y (range)
**VITAMIN B**_**12**_ **(COBALAMIN)**												
Dickey et al. (2008)	Yes	Yes	Yes	Serum vitamin B_12_ (pmol/l)	24		Mean: 242.5 (SD: 105.8)	41		Mean: 276.6 (SD: 137.1)	Not reported	1 y (min)
Fang et al. (2017)	No	No^c^	Yes	Serum vitamin B_12_ *(cut-off: 180 ng/l)*	60	2 (3)		175	3 (2)		*p* = 0.6023	2 y (min)
Kemppainen et al. (1998)	No	Yes	(?)	Serum vitamin B_12_ *(cut-off: not reported)*	37	1 (3)		3	0 (0)		Not reported	1 y
Pekki et al. (2015)	Yes	Yes	No	Serum vitamin B_12_ (pmol/l)	85		Median: 383 (Q_1_-Q_3_: 286–464)	178		Median: 341 (Q_1_-Q_3_: 269-428)	*p* = 0.153	1 y
Thornquist et al. (1993)	Yes	No^b^	No	Serum vitamin B_12_	4		Not reported^d^	9		Not reported^d^	Not reported	17 y (mean) with 15–18 y (range ?)
**VITAMIN A (RETINOL)**												
Kemppainen et al. (1998)	No	Yes	(?)	Serum vitamin A *(cut-off: not reported)*	37	5 (14)		3	0 (0)		Not reported	1 y
**VITAMIN D (CHOLECALCIFEROL)**												
Fang et al. (2017)	No	No^c^	Yes	Serum total 25-hydroxy vitamin D_2_ and D_3_ *(cut-off: 25 ng/ml)*	55	17 (31)		174	14 (8)		*p* = 0.0001 (favors recovery)	2 y (min)
Valdimarsson et al. (1994)	Yes	(?)	No	Serum 25-hydroxy vitamin D	13		Median: 52 (range: 18–91)	17		Median: 80 (range: 37-95)	*p* < 0.05 (favors recovery)	8 y and 9 y (medians for groups) with 4–14 y (range)
**IRON AND FERRITIN**												
Fang et al. (2017)	No	No^c^	Yes	Serum ferritin *(cut-off: males: 24 mcg/l, females: 11 mcg/l)*	69	7 (10)		165	21 (13)		*p* = 0.6636	2 y (min)
Kemppainen et al. (1998)	No	Yes	(?)	Serum total iron *(cut-off: not reported)*	37	15 (41)		3	0 (0)		Not reported	1 y
Kemppainen et al. (1998)	No	Yes	(?)	Serum ferritin *(cut-off: not reported)*	37	5 (14)		3	0 (0)		Not reported	1 y
Pekki et al. (2015)	Yes	Yes	No	Serum total iron (μmol/l)	∑144^a^		Median: 19.6 (Q_1_-Q_3_: 13.4–24.4)	∑144^a^		Median: 17.6 (Q_1_-Q_3_: 14.0-21.5)	*p* = 0.245	1 y
Souroujon et al. (1982)	Yes	Yes	(?)	Serum ferritin	2		Mean: 16.2 (SD: 1.38)	34		Mean: 25.7 (SD: 1.82)	Not reported	1 y
**CALCIUM**												
Fang et al. (2017)	No	No^c^	Yes	Serum total calcium *(cut-off: 8.9 mg/dl)*	65	13 (20)		164	4 (2)		*p* < 0.0001 (favors recovery)	2 y (min)
Pekki et al. (2015)	Yes	Yes	No	Serum ionized calcium (mmol/l)	∑95^a^		Median: 1.25 (Q_1_-Q_3_: 1.21-1.28)	∑95^a^		Median: 1.24 (Q_1_-Q_3_: 1.21-1.27)	*p* = 0.708	1 y
Valdimarsson et al. (1994)	Yes	(?)	No	Serum ionized calcium (mmol/l)	13		Median: 1.24 (range: 1.16-1.31)	17		Median: 1.24 (range: 1.19-1.34)	*p* = NS	8 y and 9 y (medians for groups) with 4–14 y (range)
**ZINC**												
Fang et al. (2017)	No	No^c^	Yes	serum zinc *(cut-off: 0.66 mcg/ml)*	46	18 (39)		145	20 (14)		*p* = 0.0005 (favors recovery)	2 y (min)
Kemppainen et al. (1998)	No	Yes	(?)	serum zinc *(cut-off: not reported)*	37	10 (27)		3	0 (0)		Not reported	1 y
**COPPER**												
Fang et al. (2017)	No	No^c^	Yes	Serum copper (0.75 mcg/ml)	51	0 (0)		149	4 (3)		*p* = 0.5740	2 y (min)
**HOMOCYSTEINE**												
Dickey et al. (2008)	Yes	Yes	Yes	Plasma homocysteine (μmol/l)	24		Mean: 11.4 (SD: 5.5)	41		Mean: 10.4 (SD: 2.6)	Not reported	1 y (min)

a Only the total N^0^ of pts was given.

b Parameter was measured years after the follow-up biopsy.

c Parameter was measured within 1 month of follow-up biopsy.

d *Values were within the normal range for all patients and not reported for recovery and atrophic groups separately. (?) indicates uncertainty. CeD, celiac disease; EGRAC, erythrocyte glutathione reductase activation coefficient; GFD, gluten-free diet; m, month; min, minimum; NS, non-significant; Q_1_-Q_3_, 25% and 75% quartiles; SD, standard deviation; y, year*.

The association of water-soluble vitamins and follow-up histology is understudied. We found the numerical values of vitamins B_2_ and B_6_ levels or their laboratory indicators in patients with MR and PVA but the studies did not perform statistical comparison (Dickey et al., 2008). Regarding vitamins B_12_ and B_9_ (folic acid) levels, studies found no difference between groups by histology (Pekki et al., 2015; Fang et al., 2017). No reports are available on other water-soluble vitamins.

Reports on fat-soluble vitamins are scarce. Only numerical data are available on vitamin A without statistical comparison made by the authors (Valdimarsson et al., 1994). Patients with PVA tended to have lower 25-hydroxyvitamin D level [and total calcium (Fang et al., 2017)], though serum parathyroid hormone (Valdimarsson et al., 1994; Pekki et al., 2015), ionized calcium (Valdimarsson et al., 1994; Pekki et al., 2015) and alkaline phosphatase (Valdimarsson et al., 1994) seemed similar in patients with MR and PVA.

Zinc but not copper deficiency may be associated with PVA (39 vs. 14% for zinc deficiency with PVA and MR, respectively, *p* = 0.0005) (Fang et al., 2017).

#### Anemia

Nine studies reported on hemoglobin, four studies in dichotomies (aaemic vs. non-anemic) and five as continuous data (Thornquist et al., 1993; Kemppainen et al., 1998; Ciacci et al., 2002; Cammarota et al., 2007; Kaukinen et al., 2007; Tuire et al., 2012; Pekki et al., 2015; Fang et al., 2017; Mahadev et al., 2017), of which four reported statistical comparisons (Kaukinen et al., 2007; Pekki et al., 2015; Fang et al., 2017; Mahadev et al., 2017) (Table 8). Hemoglobin levels proved to be not statistically different in two studies (Kaukinen et al., 2007; Pekki et al., 2015). Another two studies examined the frequency of anemia (Fang et al., 2017; Mahadev et al., 2017), of which one found it more frequent with PVA (22 vs. 18%, *p* = 0.04) (Mahadev et al., 2017). However, the association was not confirmed after adjusting for co-variates (*p* = 0.236).

**Table 8:** Anemia and hemoglobin levels in celiac patients with persistent villous atrophy and mucosal recovery.

**Study**	**Parameter of interest assessed prospectively?**	**Parameter of interest assessed at the time of follow-up biopsy?**	**Only strict GFD?**	**Laboratory study**	**Persistent villous atrophy**			**Mucosal recovery**			**Statistics (atrophy vs. recovery)**	**Timing of follow-up biopsy (from CeD diagnosis)**
					**Total N^**0**^ of pts**	**N^**0**^ of pts with deficiency (% of total)**	**Level**	**Total N^**0**^ of pts**	**N^**0**^ of pts with deficiency (% of total)**	**Level**		
Cammarota et al. (2007)	Yes	Yes	No	Iron-deficiency anemia *(Hb cut-off: not reported)*	49	16 (33)		16	4 (25)		Not reported	6–14 m (range)
Ciacci et al. (2002)	No	Yes	No	Hb (g/l)	93		Mean: 133	297		Not reported^a^	Not reported	7.78 y (mean)
Fang et al. (2017)	No	No^b^	Yes	anemia (*Hb cut-off: males: 135 g/l, females: 120 g/l*)	97	14 (14)		249	32 (13)		*p* = 0.7255	2 y (min)
Kaukinen et al. (2007)	Yes	Yes	Yes	Hb (g/l)	13		mean: 121 (CI: 114–128)	18		Mean: 13.2 (CI: 12.6–13.8)	*p* = NS	8 y and 10 y (medians for groups) with 3–30 y (range)
Kemppainen et al. (1998)	No	Yes	(?)	anemia *(Hb cut-off: not reported)*	37	7 (19)		3	1 (33)		Not reported	1 y
Mahadev et al. (2017)	No	Yes	Yes	anemia *(Hb cut-off: not reported)*	511	114 (22)		834	148 (18)		unadjusted *p* = 0.04, **adjusted** ***p*** **=** **0.236**^**c**^	4 y (median) with 1 y (min)
Pekki et al. (2015)	Yes	Yes	No	Hb (g/l)	∑153^f^		Median: 145 (Q_1_-Q_3_: 134–150)	∑153^f^		Median: 147 (Q_1_-Q_3_: 147-151)	*p* = 0.342	1 y
Thornquist et al. (1993)	Yes	No^d^	No	Hb	4		Not reported^e^	9		Not reported^e^	Not reported	17 y (mean) with 15–18 y (range ?)
Tuire et al. (2012)	Yes	Yes	Yes	Hb (g/l)	7		Median: 140 (range: 128–161)	170		Not reported^a^	Not reported	2–41 y (range)

Multivariate analyses are highlighted with bold.

a Values were given by Marsh 0 and 1–2 separately.

b Parameter was assessed within 1-month of follow-up biopsy.

c The analysis was adjusted for age, gender, body mass index, duration of GFD, medications, laboratory tests, and symptoms.

d Parameter was measured years after the follow-up biopsy.

e *Values by recovery and atrophy were not reported separately. (?) indicates uncertainty. CeD, celiac disease; CI: confidence interval; GFD, gluten-free diet; Hb, hemoglobin; m, month; min, minimum; NS, non-significant; Q_1_-Q_3_, 25% and 75% quartiles; SD, standard deviation; y, year*.

#### Body Mass Index

Of the six studies reporting body mass index, three provided statistical evidence on having no significant difference between groups while the other three reported numerical values without analysis (Valdimarsson et al., 1994; Kaukinen et al., 2007; Dickey et al., 2008; Tuire et al., 2012; Pekki et al., 2015; Cornell et al., 2016) (Table 9).

**Table 9:** Body mass index in celiac patients with persistent villous atrophy and mucosal recovery.

**Study**	**Parameter of interest assessed prospectively?**	**Parameter of interest assessed at the time of follow-up biopsy?**	**Only strict GFD?**	**Persistent villous atrophy**		**Mucosal recovery**		**Statistics (atrophy vs. recovery)**	**Time between the diagnosis of CeD and follow-up biopsy**
				**Total N^**0**^ of pts**	**Body mass index (kg/m^**2**^)**	**Total N^**0**^ of pts**	**Body mass index (kg/m^**2**^)**		
Cornell et al. (2016)	Yes	Yes	(?)	9	Mean: 22.72 (SD: 3.10)	11	Mean: 24.26 (SD: 3.70)	Not reported	4 m-36 y (range)
Dickey et al. (2008)	Yes	Yes	Yes	24	Mean: 25.7 (SD: 4.7)	41	Mean: 24.9 (SD: 3.9)	Not reported	1 y (min)
Kaukinen et al. (2007)	Yes	Yes	Yes	13	Mean: 23 (range: 18–29)	18	Mean: 26 (range: 19–35)	*p* = NS	8 y and 10 y (medians for groups) with 3–30 y (range)
Pekki et al. (2015)	Yes	Yes	No	∑136^a^	Median: 23.9 (Q_1_-Q_3_: 22.0–27.3)	∑136^a^	Median: 24.3 (Q_1_-Q_3_: 22.1–27.1)	*p* = 0.823	1 y
Tuire et al. (2012)	Yes	Yes	Yes	7	Median: 24 (range: 21–29)	170	Not reported^b^	Not reported	2–41 y (range)
Valdimarsson et al. (1994)	Yes	(?)	No	13	Median: 23.7 (range: 17–29)	17	Median: 23.6 (range: 19–30)	*p* = NS	8 y and 9 y (medians for groups) with 4–14 y (range)

Mahadev et al. (2017) reported body mass index categories (not shown). Although body mass index was associated with villous atrophy in univariate analysis (p = 0.014), it proved to be independent of histology after adjustment for covariates (p = NS for all categories).

a The number of patients were not reported separately for atrophy and recovery groups.

b *Values were given by Marsh 0 and 1–2 separately. (?) indicates uncertainty. CeD, celiac disease; GFD, gluten-free diet; m, month; min, minimum; NS, non-significant; Q_1_-Q_3_, 25% and 75% quartiles; SD, standard deviation; y, year*.

#### Malignant Tumors and Other Co-morbid Conditions

Four studies reported the overall rate of malignant tumors during follow-up (Kaukinen et al., 2007; Tuire et al., 2012; Pekki et al., 2015, 2017), two of which performed univariate analysis where event rates were 4.5 vs. 5.1% (*p* = 0.762) and 11.3 vs. 6% (*p* = 0.116) with PVA vs. MR, respectively (Pekki et al., 2015, 2017) (Table 10).

**Table 10:** Malignant tumors in celiac patients with persistent villous atrophy and mucosal recovery.

**Study**	**Only strict GFD?**	**Malignant tumors**	**N**^****0****^ **of cases/Total** **(%)**		**Statistics**	**Timing of follow-up biopsy** **(from diagnosis of CeD)**	**Follow-up period**
			**Persistent villous atrophy**	**Mucosal recovery**			
Kaukinen et al. (2007)	Yes	Any malignant tumor^a^	3 cases/13 pts (23.1)	0 cases/18 pts (0.0)	Not reported	8 y and 10 y (medians for groups) (range: 3–30 y)	4–5 years from follow-up biopsy
Lebwohl et al. (2013b)	No	Any lymphoma	41 cases/4317 pts (0.9)	12 cases/3308 pts (0.4)	**HR** **=** **2.26 (CI: 2.28–4.34)**^**d**^	1.3 (median) (range: 6 m−5 y)	10.6 y (median) from diagnosis of CeD and 8.9 y (median) from follow-up biopsy
		Any non-Hodgkin lymphoma	34 cases/4317 pts (0.8)	8 cases/3308 pts (0.2)	**HR** **=** **2.82** **(CI: 1.29–6.17)**^**d**^		
		Any B-cell lymphoma	4 cases/4317 pts (<0.1)	3 cases/3308 pts (<0.1)	**HR** **=** **0.97** **(CI: 0.21–4.49)**^**d**^		
		Any T-cell lymphoma	10 cases/4317 pts (0.2)	2 cases/3308 pts (<0.1)	**HR** **=** **3.51** **(CI: 0.75–16.34)**^**d**^		
Pekki et al. (2015)	No	Any malignant tumor	9 cases/200 pts (4.5)	14 cases/276 pts (5.1)	*p* = 0.762	1 y	8 y (median) from diagnosis of CeD
Pekki et al. (2017)	No	Any malignant tumor^b^	8 cases/71 pts (11.3)	8 cases/134 pts (6.0)	*p* = 0.116	1 y	5 y (median) from diagnosis of CeD
		Any lymphoma	1 case/71 pts (1.4)	2 cases/134 pts (1.5)	*p* = 0.968		
Tuire et al. (2012)	Yes	Any malignant tumor^c^	0 cases/7 pts (0.0)	4 cases/170 pts (2.4)	Not reported	7 y, 9 y, and 10 y (medians for groups) (range: 2-41 y)	From diagnosis of CeD until follow-up biopsy

Multivariate analyses are highlighted with bold.

a Cases included T-cell lymphoma, small bowel adenocarcinoma, and carcinoid tumor;

b Cases included the uterus, breast, lung, pancreas, urinary bladder, and prostate tumors; lymphomas, and sarcomas.

c Cases included breast, uterine, and prostate cancer; and lymphoma.

d *The analysis was adjusted for age, sex, year of follow-up biopsy, education, and duration of celiac disease at the time of follow-up biopsy. CeD, celiac disease; CI, confidence interval; GFD, gluten-free diet; HR, hazard ratio; pts, patients; m, month; y, year*.

Incidence of lymphomas was 1.4 vs. 1.6% (*p* = 0.968) with PVA vs. MR in univariate analysis (Pekki et al., 2015). In a registry analysis, PVA had an adjusted HR of 2.26 (CI: 1.18–4.34) for the overall rate of lymphomas, being true for the subgroup of non-Hodgkin lymphomas but not for the subset of T-cell lymphomas (Lebwohl et al., 2013b) (Table 10).

Sporadic records concerned other co-morbid conditions. Cardiovascular diseases including heart failure and atrial fibrillation (Lebwohl et al., 2015a), hypertension (Mahadev et al., 2017), serious infections (including sepsis, streptococcal, pneumococcal, influenza, herpes zoster, and *Clostridium difficile* infections) (Emilsson et al., 2018) and adverse pregnancy outcomes (Lebwohl et al., 2015b) were not influenced by follow-up histology. However, there was a significant reduction in the frequency of respiratory and dermatological diseases (Pekki et al., 2017) and in the rate of elevated aspartate and alanine transaminase levels with MR (Mahadev et al., 2017). In contrast, MR predisposed to developing anxiety and depression (Ludvigsson et al., 2018) as well as epilepsy (Kurien et al., 2018). The effect of follow-up histology on immune-mediated co-morbidities including dermatitis herpetiformis is severely understudied (Valdimarsson et al., 1994; Tuire et al., 2012; Mahadev et al., 2017).

#### All-Cause and Cause-Specific Mortality

Four studies reported all-cause mortality during follow-up (Kaukinen et al., 2007; Rubio-Tapia et al., 2010; Lebwohl et al., 2013a; Pekki et al., 2015), two of which adjusted the analysis for significant co-variates (Rubio-Tapia et al., 2010; Lebwohl et al., 2013a). In this regard, MR seemed to be protective in a small retrospective study with a HR of 0.13 (CI: 0.02–1.06) (Rubio-Tapia et al., 2010), not confirmed by a nationwide registry analysis (HR = 1.0, CI: 0.86–1.19 for PVA) (Lebwohl et al., 2013a). In the latter study, results were consistent on cardiovascular, tumor-related and respiratory deaths: the beneficial effect of MR was not confirmed (Lebwohl et al., 2013a) (Table 11).

**Table 11:** Studies reporting on mortality in celiac patients with persistent villous atrophy and mucosal recovery.

**Study**	**Only strict GFD?**	**Mortality**	**N**^****0****^ **of cases/Total** **(%)**		**Statistics (atrophy vs. recovery)**	**Timing of follow-up biopsy** **(from diagnosis of CeD)**	**Follow-up period**
			**Persistent villous atrophy**	**Mucosal recovery**			
Kaukinen et al. (2007)	Yes	All-cause	2 cases/13 pts (15.4)	1 case/18 pts (5.6)	Not reported	8 y and 10 y (medians for groups) (range: 3–30 y)	4–5 years from follow-up biopsy
Pekki et al. (2015)	No	All-cause	10 cases/71 pts (14.1)	12 cases/134 pts (9.0)	*p* = 0.259	1 y	8 y (median) from diagnosis of CeD
Rubio-Tapia et al. (2010)	No	All-cause	10 cases/117 pts (8.5)	1 case/124 pts (0.8)	**HR** **=** **0.13** **(CI: 0.02–1.06)**^**a**^ ***p*** **=** **0.06 (favors recovery)**	Min: 5 m	10 y from diagnosis of CeD
Lebwohl et al. (2013a)	No	All-cause Cardiovascular Malignancy Respiratory	Unknown number of 3,317 pts^b^	Unknown number of 4,331 pts^b^	HR = 1.37 (CI: 1.16–1.62)^c^ (favors recovery); **HR** **=** **1.01** **(CI: 0.86–1.19) after adjustment**^**d**^	1.3 (median) (range: 6 m−5 y)	11.5 y (median) from diagnosis of CeD and 9.9 y (median) from follow-up biopsy
					**HR** **=** **1.03** **(CI: 0.76-1.38)**^**d**^		
					**HR** **=** **1.20** **(CI: 0.88-1.66)**^**d**^		
					**HR** **=** **0.78** **(CI: 0.41-1.48)**^**d**^		

Multivariate analyses are highlighted with bold.

a The analysis was adjusted for age and gender.

b Total mortality of the cohort of patients was 8.0%.

c The analysis was unadjusted.

d *The analysis was adjusted for age, gender, calendar period of diagnosis, education level, and length of follow-up. CeD, celiac disease; CI, confidence interval; GFD, gluten-free diet; HR, hazard ratio; pts, patients; m, month; y, year*.

## Discussion

### Summary of Findings

Whether the follow-up biopsy in asymptomatic CeD is needed is uncertain, and, to date, no systematic review has addressed this question. Achieving MR is a desirable goal of treatment; however, findings of reports which investigated its beneficial effects on disease course are inconsistent. In line with our previous findings, MR rates ranged from 9 to 97% across the studies included (Szakacs et al., 2017). The fact that a considerable fraction of CeD patients does not achieve MR underlines the importance of investigating the potential prognostic role of follow-up histology.

One important conclusion of this review is that many asymptomatic patients do not achieve MR, and vice versa, many symptomatic patients do achieve MR. Analogously, MR cannot guarantee the symptoms to disappear, whereas PVA is often associated with persisting symptoms, even on a long-term follow-up exceeding 1 year (Tables 2, 3). Persisting symptoms may indicate poor dietary adherence (Abdulkarim et al., 2002; Leffler et al., 2007; Haere et al., 2016) or gluten-independent food intolerance (Carroccio et al., 2008) but other diseases, such as pancreatic insufficiency or small intestinal bacterial overgrowth, might be in the background (Fine et al., 1997). Noteworthy that a few studies included patients with questionable adherence to gluten-free diet, although a top-quality study established a straight relationship between persisting symptoms and PVA in co-variate-adjusted analysis of patients with good adherence (Carroccio et al., 2008). Studies used various definitions for assessing symptoms; some did not report how symptoms were specified at all. Although a smaller study favored MR regarding a set of individual symptoms (Carroccio et al., 2008), the most extensive study including only symptomatic patients found significant associations in univariate analysis (with an unexpected inverse association between PVA and heartburn). Interestingly, all associations proved to be non-significant after adjusting for co-variates (Mahadev et al., 2017). Self-reporting of subjective complaints may contribute to the discrepant results. Use of symptom scales (Svedlund et al., 1988) provided a comparable measure with homogenous results: none of the studies attributed higher scores for those patients with PVA compared to those with MR (Table 4). The same association applies to quality of life; however, based on small sets of patients (Table 4). Altogether, the diagnostic accuracy of persisting symptoms seems insufficient for indicating a follow-up biopsy. Evidence coming from the two prospective studies is not enough to decide whether PVA precisely predicts the long-term persistence of symptoms (Pekki et al., 2015, 2017) (Table 4).

PVA appears to be associated with radiologically detected metabolic bone disease in univariate analysis (Table 5), which translates into an increased risk of hip fractures (as an independent predictor) but not into that of the overall fractures (Table 6). Calcium malabsorption (Fang et al., 2017) together with vitamin D deficiency (Valdimarsson et al., 1994; Fang et al., 2017) are likely to be causative factors. Length of follow-up (not standardized among studies) may be insufficient for restoring bone mineral density (Pekki et al., 2015), especially in those with severe bone impairment at diagnosis. Body mass index, as an important co-variate, seems to be independent of mucosal status (Table 9).

Laboratory studies describing vitamin and mineral levels tend to recover on an adequate diet. Patients on a long-term strict gluten-free diet may suffer from vitamin deficiencies (Hallert et al., 2002, 2009), this phenomenon might be explained by the vitamin- and micronutrient-poor nature of the gluten-free diet as compared to a balanced gluten-containing diet (Thompson, 1999). None of the studies adjusted for dietary adherence and vitamin supplementation which might counteract malabsorption and masked the effects of PVA. Variability in clinical and histological severity at diagnosis might delay villous restitution, thereby affecting vitamin status (not taken into account in the studies) (Kemppainen et al., 1998). Besides, improvement of histology might lag behind the recovery of laboratory values (Pekki et al., 2015), although the length of follow-up exceeded 1 year in all studies (Table 7).

Regarding mortality, the adjusted HRs calculated in the individual studies attributed a neutral effect to MR and PVA (Table 11) (Rubio-Tapia et al., 2010; Lebwohl et al., 2013a). Findings were similar on malignant tumors, except in certain lymphoproliferative diseases based on an extensive registry analysis (noteworthy that data were not adjusted for dietary adherence) (Lebwohl et al., 2013b). A possible explanation could be that the increment in rates of mortality and tumors with PVA was counteracted by a decreased risk of ovarian, endometrial and breast cancer, likely due to lower body weight and/or earlier menopause (Ludvigsson et al., 2012; Lebwohl et al., 2013a).

### Strengths and Limitations

Our study has several strengths. The question we raised is unique without known previous systematic review. We carried out an extensive systematic search with high coverage of patient-important outcomes in a transparent manner by independent (and pre-trained) investigators with good inter-rater agreement. The quality of the included papers was rigorously assessed.

**Figure 2:**
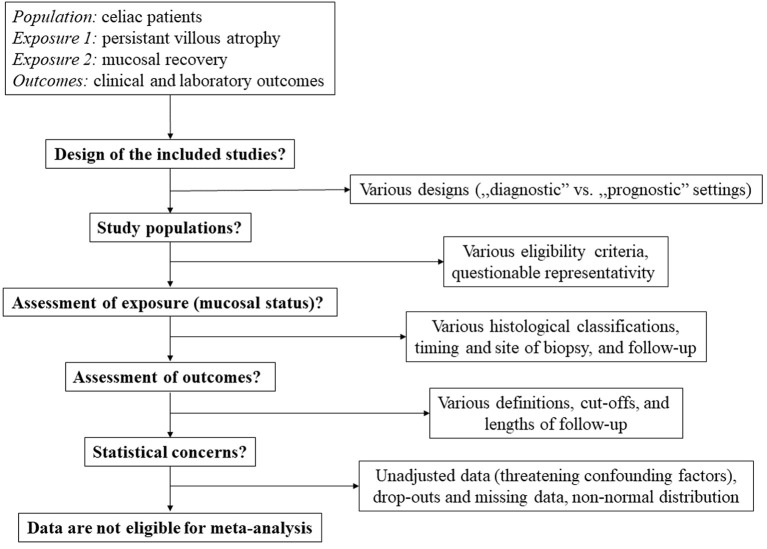
Decision-making algorithm. Based on several arguments, we decided not to perform a meta-analysis.

Although the amount of data presented in the tables would be enough for meta-analytical aggregations that we intended to do initially (as declared in the protocol of the work, see the PROSPERO record); finally, our team decided not to do so because of several concerns. The decision-making algorithm is presented in Figure 2.

We are aware that the evidence suffers from several limitations. (1) Most studies did not recruit patients immediately after the diagnosis of CeD (29/31, 94%), did not take the follow-up biopsy after recruitment (23/31, 74%) and did not assess the disease course prospectively. (2) Although the diagnosis of CeD was mainly biopsy-proven, only two papers included newly diagnosed cases and observed them longitudinally until the follow-up biopsy. (3) Details of histological sampling and processing (e.g., sampling site, number of tissue samples, orientation, staining) were not consistently reported across papers, while the procedures often deviated from recent gold standards (e.g., samples were taken from the jejunum, histological classification systems were not specified). Similarly, the definition of villous atrophy varied across the studies. (4) Observational studies are vulnerable to bias: in registry analyses, data of the deceased were often missing (survivorship bias), attendance to regular control visits was incomplete (attrition bias), baseline differences between groups of patients with MR and those with PVA were rarely balanced with co-variate-adjusted analyses (selection bias), study samples were not taken consistently from the general CeD population with consecutive recruitment (selection bias), and investigators assessing the outcomes were rarely blind to mucosal status (performance bias, detection bias). (5) Most evidence came from studies focusing on adults while an inverse association between age and rate of MR is well-known (Szakacs et al., 2017). (6) A strict gluten-free diet was not a criterion in several studies, the length of diet varied. (7) Publication bias cannot be reliably assessed in systematic reviews.

## Conclusion

### Implications for Practice

The results of publications on the prognostic role of follow-up histology (that is, MR and PVA) are not in agreement. Some adverse outcomes (e.g., persistent symptoms and metabolic bone disease) may be more common with PVA; however, achieving MR alone cannot guarantee a favorable clinical course to our current knowledge. The question as to whether taking a follow-up biopsy is beneficial has remained a matter of debate.

### Implications for Research

With a view to the future, prospective cohort studies are urged to be organized to collect decisive evidence on the prognostic role of follow-up histology.

## Data Availability Statement

All datasets analyzed this study are included in the manuscript/supplementary files.

## Author Contributions

ZS and JB conceptualized the study. AM, DD, VB, LS, AV, and RH collected the data. NG performed the formal analysis and designed the figures. ZS assessed the quality of studies. ZG, DC, and MS interpreted the results. ÁV, ZS, JB, and BE drafted the manuscript. ÁV and PH supervised the work. All authors approved the final draft submitted, involved in the study design, edited, read and approved the final manuscript.

### Conflict of Interest

The authors declare that the research was conducted in the absence of any commercial or financial relationships that could be construed as a potential conflict of interest.

## Abbreviations

SF-36: 36-Item Short Form Health Survey
CeD: celiac disease
GSRS: Gastrointestinal Symptom Rating Scale
HR: hazard ratio
IBS: irritable bowel syndrome
MR: mucosal recovery
NS: non-significant
OR: odds ratio
PGWB: Psychological General Well-Being Index
PVA: persistent villous atrophy.
